# Exploratory Analysis of Skeletal Muscle Architecture and Force–Time Strategy Under External Load in Collegiate Basketball Players

**DOI:** 10.3390/jfmk11030246

**Published:** 2026-06-24

**Authors:** Chieh-Ying Chiang, Tzu-Han Chan, Yi-Cheng Wu, Sung-Kai Lin

**Affiliations:** 1 Department of Sports Training Science-Combats, National Taiwan Sport University, Taoyuan 33325, Taiwan; 2 Department of Sports Science Research, Taiwan Institute of Sports Science, Kaohsiung 81343, Taiwan; 3 Department of Athletic Training and Health, National Taiwan Sport University, Taoyuan 33325, Taiwan; 1140601@ntsu.edu.tw; 4 Department of Sport Medicine, Landseed International Hospital, Taoyuan 32449, Taiwan; keanu.firefox@gmail.com (Y.-C.W.); linedbeng@landseed.com.tw (S.-K.L.)

**Keywords:** loaded retention, rate of force development, force–time strategy, stretch–shortening cycle, neuromuscular profiling, ultrasonography

## Abstract

**Objectives**: Skeletal muscle architecture (SMA) defines the mechanical limits of force production. However, its associations with force–time strategy under externally loaded conditions have received little research attention. This exploratory study examined associations between vastus lateralis (VL) and lateral gastrocnemius (LG) architecture and force–time strategy, jump-height retention, and stretch–shortening cycle (SSC) transfer-efficiency in collegiate basketball players. **Methods**: Seventeen collegiate male basketball players completed B-mode ultrasonographic assessment of VL and LG architecture, including muscle thickness, pennation angle (PA), and fascicle length. Athletes performed the squat jump (SJ), loaded squat jump (LSJ), countermovement jump (CMJ), and loaded countermovement jump (LCMJ) on force platforms, with a 20 kg external load applied for loaded conditions. Loaded retention, defined as the percentage of jump height preserved under load, was proposed as a unified construct. Pearson’s correlations were calculated, with Benjamini–Hochberg false discovery rate (FDR) corrections applied within predefined functional groups and pooled across morphology-sensitive correlations. **Results**: LG PA showed a large negative association with LCMJ rate of force development (*r* = −0.68 [−0.87, −0.30]) and a large positive association with LCMJ time to peak force (r = 0.68 [0.29, 0.87]), both surviving within-group FDR correction. VL PA was associated with eccentric acceleration time and concentric time across jump conditions (*r* = 0.52 to 0.61), interpreted as exploratory. Transfer-efficiency indices showed no significant associations with SMA, except for the LCMJ/LSJ concentric time ratio, which showed a moderate negative association with LG PA (*r* = −0.49 [−0.79, −0.01]). **Conclusions**: Resting muscle architecture was associated with the temporal and rate characteristics of force expression under load, rather than with the gross preservation of jump height. Integrating architectural assessment with loaded force–time profiling warrants further investigation as a means of characterizing individual force-development strategies.

## 1. Introduction

Skeletal muscle architecture (SMA), encompassing muscle thickness (MT), pennation angle (PA), and fascicle length (FL), defines the mechanical limits of muscular force production [1,2]. Greater MT and PA are associated with a larger physiological cross-sectional area, facilitating maximal force production, whereas longer FL is associated with higher shortening velocities and improved compliance [3,4]. The applied relevance of SMA in athletic populations lies primarily in its influence on the temporal expression and rate of force development (RFD) [5].

Vertical jump performance is widely used to characterize neuromuscular function in athletes [6,7]. However, evidence linking static SMA measures to maximal jump height has been inconsistent. A systematic review and meta-analysis reported that 74% of associations between SMA and maximal jump height did not reach statistical significance [8], and similar findings have emerged from ultrasound-based studies of the vastus lateralis (VL) and lateral gastrocnemius (LG) in jumping athletes [9,10]. These observations suggest that static morphology is insufficient to predict the neuromuscular coordination required for jumping [11]. B-mode ultrasonography may therefore be better suited to examining how architectural characteristics relate to the mechanical strategies through which force is expressed, rather than predicting absolute jump outcomes.

Architectural gearing theory provides a directional framework for SMA’s influence on force–time strategy [3,8]. A greater PA enhances absolute force capacity through a pennation-based mechanical advantage, whereas the same architectural feature may constrain the rate of force transmission to the tendon during dynamic stretch–shortening cycle (SSC) actions [2,3]. Within this framework, pennation governs a trade-off between maximal force capacity and the speed of force transmission. Consequently, the temporal cost of this structural arrangement becomes more pronounced when external inertia extends the braking phase, demanding a rapid transition from yielding to concentric force production. Specifically, greater LG PA would be expected to associate with lower RFD and a longer time to peak force, particularly when external inertia extends the braking phase. The VL, with its unipennate architecture and typically greater PA, is expected to modulate the temporal distribution of force across the concentric phase [12].

Elite court sports involve frequent high-inertia actions, physical contact, and resisted accelerations, which limit the ecological validity of conventional unloaded jump assessments [13] and emphasize the importance of retaining jump performance under external loads. In professional basketball, force–time characteristics relate more closely to on-court efficiency than jump height alone [14,15], further motivating attention to the temporal and rate features of force expression. Furthermore, the eccentric utilization ratio (EUR), the most commonly used SSC efficiency index, provides limited mechanical resolution for characterizing these strategies, as it is derived from jump-height ratios rather than rate or temporal characteristics [16,17,18]. Building on prior weighted-jump research [19,20], loaded retention is here proposed as a unified construct, defined as the percentage of jump height preserved under a fixed external load, which may offer a more ecologically valid performance metric for contact-sport populations. Unlike single-condition jump height, loaded retention expresses the proportion of unloaded output maintained when external inertia is added. This proportional framing separates load tolerance from absolute jump capacity. The construct therefore targets a performance quality that conventional jump-height monitoring leaves unmeasured. Despite the recognized importance of both SMA and loaded jump performance, the associations between resting muscle architecture and force–time strategy under externally loaded conditions have received little research attention.

Consequently, this study aimed to investigate the associations between resting SMA of the VL and LG and force–time strategy under loaded and unloaded vertical jump conditions in collegiate basketball players. A secondary, exploratory aim was to examine the relationships between SMA and jump-height retention and SSC transfer-efficiency indices, for which no directional hypotheses were specified. It was hypothesized that greater LG PA would be associated with lower LCMJ RFD and longer time to peak force. VL PA was hypothesized to relate to the temporal organization of force expression.

## 2. Materials and Methods

### 2.1. Study Design

An exploratory, cross-sectional design was used, involving resting ultrasonographic assessment of lower-body muscle architecture followed by multi-condition vertical jump testing on a set of force platforms within a single session. Jump conditions comprised the countermovement jump (CMJ), squat jump (SJ), loaded countermovement jump (LCMJ), and loaded squat jump (LSJ), with a 20 kg external load applied for all loaded conditions [20].

### 2.2. Participants

Seventeen collegiate male basketball players (age: 20.4 ± 1.2 years; height: 184.6 ± 8.3 cm; body mass: 82.6 ± 12.0 kg; body mass index: 24.6 ± 1.8 kg·m^−2^) with a minimum of six years of basketball-specific training experience participated in this study. Inclusion required active collegiate-level competition; participants were excluded if they reported any current lower-body musculoskeletal injury or had undergone orthopedic surgery within the preceding 12 months. All participants provided written informed consent, and the study was approved by the Institutional Review Board at Fu Jen Catholic University (C108178) and conducted in accordance with the Declaration of Helsinki.

### 2.3. Vertical Jump Testing

#### 2.3.1. Warm-Up and Familiarization

The warm-up consisted of five minutes of light aerobic activity, followed by dynamic lower-body movements including bodyweight squats, walking lunges, and leg swings. Participants were then familiarized with each jump condition through two submaximal practice trials at approximately 50% and 75% of perceived maximal effort.

#### 2.3.2. Jump Conditions

Four jump conditions were assessed: SJ, CMJ, LSJ, and LCMJ, consistent with procedures previously reported for weighted and unweighted vertical jump assessment [20]. For loaded conditions, participants held a bar across the upper back, positioned between the seventh cervical and third thoracic vertebrae. For unloaded conditions, a lightweight wooden stick (<1 kg) was used; for loaded conditions, a standard 20 kg barbell was used [20].

#### 2.3.3. Squat Jump

The SJ and LSJ were initiated from a standardized position with the knees flexed to approximately 90°, verified using a hand-held goniometer. Participants held the bottom position for three seconds before jumping to eliminate the stretch–shortening cycle contribution [20].

#### 2.3.4. Countermovement Jump

The CMJ and LCMJ were initiated from an upright standing position with a self-selected countermovement depth. Participants were instructed to jump as high and as quickly as possible without pausing at the bottom of the countermovement [20].

#### 2.3.5. Testing Protocol

Testing began with the SJ conditions (SJ followed by LSJ), and the CMJ conditions (CMJ followed by LCMJ) were completed thereafter. A three-minute rest period was provided between conditions, and a one-minute rest was given between individual trials. Two maximal effort trials were performed for each condition, and the mean of both trials was used for analysis [20].

#### 2.3.6. Instrumentation and Data Processing

All jumps were performed on two force platforms (9260AA, Kistler, Winterthur, Switzerland), sampling at 1000 Hz. An analog-to-digital converter (5695B, Kistler, Winterthur, Switzerland) was interfaced with the platforms for data acquisition and signal processing using Bioware software (2812A, Kistler, Winterthur, Switzerland). Raw vertical force–time data were recorded and stored, subsequently exported as text files, and analyzed using a custom-designed Microsoft Excel spreadsheet (version 2016; Microsoft Inc., Redmond, WA, USA). All force–time variables were derived from each jump condition in accordance with a previously described analytical framework [21].

### 2.4. Skeletal Muscle Architecture Assessment

#### 2.4.1. Ultrasonographic Imaging

Resting lower-body muscle architecture was assessed using a B-mode ultrasonography system (E-CUBE 8 DIAMOND, Alpinion Medical Systems, Seoul, Republic of Korea) with a 3–12 MHz linear array transducer. All images were obtained by a single physician. Two images were recorded per muscle per limb, and values from the left and right limbs were averaged, consistent with previously established procedures [4,22]. Because all jump tasks were bilateral, this averaging provides a composite measure of overall lower-body structure and reduces the influence of unilateral measurement error.

#### 2.4.2. Vastus Lateralis (VL)

Participants were placed in a supine position with the lower limbs relaxed and fully extended. The probe was positioned longitudinally at 50% of the distance between the greater trochanter and the lateral epicondyle of the femur [4,22]. Minimal probe pressure was applied to avoid compression of the underlying muscle tissue.

#### 2.4.3. Lateral Gastrocnemius (LG)

Participants were placed in a prone position with the ankle in a neutral, relaxed position. The probe was positioned longitudinally at two-thirds of the distance between the lateral epicondyle of the femur and the lateral malleolus [4,22].

#### 2.4.4. Image Analysis

All images were analyzed using ImageJ software (version 1.53v, National Institutes of Health, Bethesda, MD, USA) by the same physician who obtained the images. MT was defined as the perpendicular distance between the superficial and deep aponeuroses. PA was defined as the angle between the muscle fascicle and the deep aponeurosis. FL was calculated as FL = MT × (sin PA)^−1^ [22]. This geometric derivation provides an estimate of fascicle length rather than a direct measurement.

### 2.5. Statistical Analysis

The study was exploratory in nature, consistent with recent recommendations for exploratory research in sport and exercise science [23]. Because the sample size was fixed, a sensitivity power analysis was performed using G*Power 3.1 (Heinrich Heine University Düsseldorf, Düsseldorf, Germany) for a bivariate normal correlation model. With n = 17, α = 0.05 (two-tailed), and power = 0.80, the minimum detectable bivariate correlation was |ρ| = 0.62. Statistical analyses were performed using SPSS 22.0 (IBM Corp., Armonk, NY, USA), with the Benjamini–Hochberg false discovery rate applied to the correlation *p* values using a custom script written in Python (version 3.12, Python Software Foundation, Wilmington, DE, USA).

Descriptive statistics, including means and standard deviations (SD), were calculated for all variables. The assumption of normality was confirmed using the Shapiro–Wilk test (all *p* > 0.05). Statistical significance was set at *p* < 0.05. Reliability of force–time variables across all four conditions, and of muscle architecture variables (MT, PA, and FL for both VL and LG), was assessed using the intraclass correlation coefficient (ICC; two-way mixed-effects model, absolute agreement) and the coefficient of variation (CV); 95% confidence intervals (CIs) and the standard error of measurement (SEM) were additionally calculated for force–time variables. Variables that did not meet acceptable thresholds (ICC < 0.70 [24] or CV > 10% [25]) were excluded.

Among the reliability-retained variables, those with a mechanistic basis for association with architectural properties were identified as morphology-sensitive and organized into three predefined functional groups before the correlation analyses (Table 1): loaded force development, force-velocity expression, and temporal force-expression. Conventional absolute outcomes were retained for supplementary analysis (Supplementary Tables S1 and S2). The resulting data-driven variable set is asymmetric across conditions and exhibits sample specificity [23]; for example, rate of force development (RFD) is represented only for the LCMJ because values from the remaining conditions did not meet the reliability threshold.

Paired-samples *t* tests compared loaded versus unloaded conditions, with effect sizes reported as Hedges’ *g* and 95% CIs. Transfer-efficiency indices were calculated as numerator/denominator × 100 and averaged. Pearson correlations examined associations between muscle architecture variables and force–time outcomes and transfer-efficiency indices, with correlation coefficients reported with 95% CIs. Correlation magnitudes were interpreted as small (*r* = 0.10), moderate (*r* = 0.30), large (*r* = 0.50), very large (*r* = 0.70), and extremely large (*r* = 0.90), and Hedges’ *g* as small (0.20), moderate (0.60), large (1.20), very large (2.0), and extremely large (4.0) [25].

Benjamini–Hochberg false discovery rate (FDR) corrections [28] were applied to characterize the multiplicity-adjusted strength of associations within each of the three predefined functional groups and pooled across all 60 morphology-sensitive correlations. Given the exploratory nature of this study and the modest sample size, inferences were guided by the magnitude and 95% CIs of the effect estimates. Both within-group and pooled corrections are reported to provide a fully transparent evaluation of the associations.

## 3. Results

Ultrasound-derived architectural variables (MT, PA, and FL for both VL and LG) showed excellent reliability (all CVs < 5%, ICCs> 0.98). Selected morphology-sensitive variables and transfer-efficiency indices showed acceptable to excellent reliability (CV = 2.76–9.00%, ICC = 0.80–0.99; Table 2 and Table 3).

### 3.1. Loaded Versus Unloaded Comparisons

**Figure 1:**
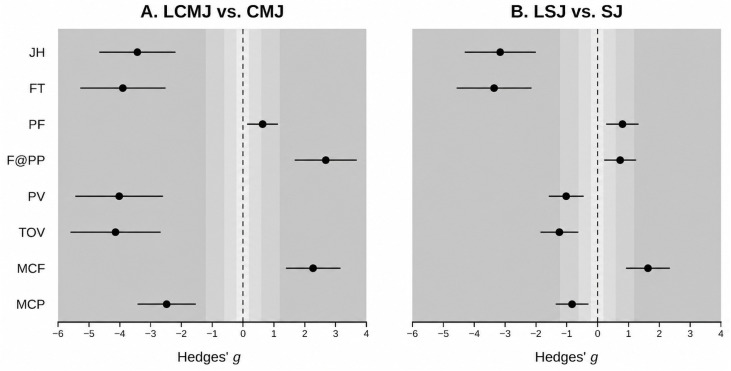
Effect sizes for loaded versus unloaded jump comparisons. Panel (**A**) shows the comparison between the loaded countermovement jump (LCMJ) and countermovement jump (CMJ), and Panel (**B**) shows the comparison between the loaded squat jump (LSJ) and squat jump (SJ). Points represent Hedges’ g, and horizontal lines represent 95% confidence intervals. Negative values indicate lower values in the loaded condition, whereas positive values indicate higher values in the loaded condition. JH = jump height; FT = flight time; PF = peak force; F@PP = force at peak power; PV = peak velocity; TOV = take-off velocity; MCF = mean concentric force; MCP = mean concentric power.

Compared with CMJ, LCMJ produced significantly lower jump height, flight time, peak velocity, take-off velocity, and mean concentric power (all *p* < 0.01, *g* = −4.14 to −2.48). Peak force, force at peak power, and mean concentric force were significantly greater under loaded conditions (all *p* ≤ 0.02, *g* = 0.63 to 2.68). Similar effects were observed when comparing LSJ with SJ. Jump height, flight time, peak velocity, take-off velocity, and mean concentric power were significantly lower under load (all *p* < 0.01, *g* = −3.36 to −0.83). Force outcomes were significantly greater under loaded conditions (*p* ≤ 0.01, *g* = 0.73 to 1.63; Figure 1; Table 4).

### 3.2. Skeletal Muscle Architecture and Morphology-Sensitive Force–Time Outcomes

LG architecture variables were associated with loaded force-development and force-velocity outcomes, whereas VL architecture variables were associated with temporal force-expression outcomes (Table 5). In contrast, LG architecture variables showed no significant associations with temporal force-expression outcomes, and VL architecture variables showed no significant associations with loaded force-development or force-velocity outcomes (Table 5).

LG PA showed a large negative association with LCMJ rate of force development (*r* = −0.68 [−0.87, −0.30], *p* = 0.003; within-group FDR-adjusted *p* = 0.016; pooled FDR-adjusted *p* = 0.080) and a large positive association with LCMJ time to peak force (*r* = 0.68 [0.29, 0.87], *p* = 0.003; within-group FDR-adjusted *p* = 0.016; pooled FDR-adjusted *p* = 0.080). LG PA also showed large negative associations with force-velocity area in the CMJ (*r* = −0.58 [−0.83, −0.14], *p* = 0.01) and LCMJ (*r* = −0.50 [−0.79, −0.03], *p* = 0.04). LG MT was associated with LCMJ RFD (*r* = −0.58 [−0.83, −0.13], *p* = 0.015; within-group FDR-adjusted *p* = 0.059; pooled FDR-adjusted *p* = 0.147) and LCMJ time to peak force (*r* = 0.53 [0.07, 0.81], *p* = 0.029; within-group FDR-adjusted *p* = 0.086; pooled FDR-adjusted *p* = 0.156). These LG MT associations were interpreted as exploratory.

**Figure 2:**
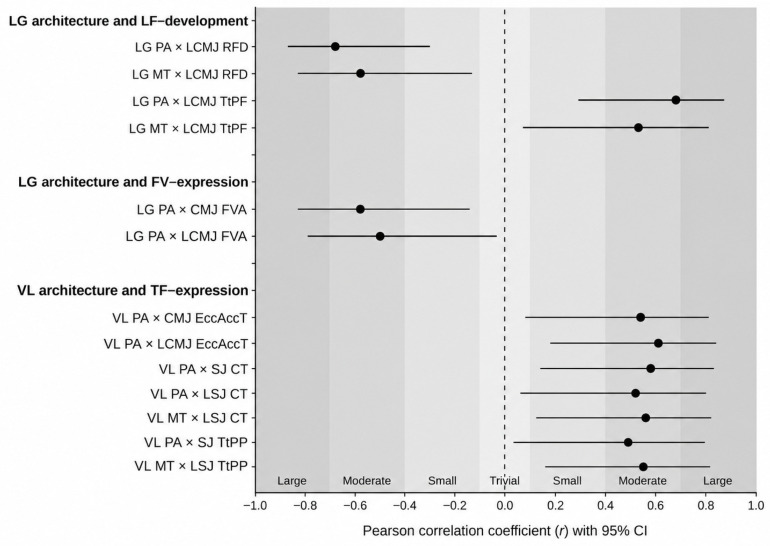
Selected associations between skeletal muscle architecture and morphology-sensitive force–time outcomes. Points indicate Pearson correlation coefficients (*r*), and horizontal lines indicate 95% confidence intervals. Selected statistically significant associations are presented. Only the associations between LG PA and LCMJ rate of force development and time to peak force remained significant after within-group FDR correction; the remaining associations should be interpreted as exploratory. LG = lateral gastrocnemius; VL = vastus lateralis; PA = pennation angle; MT = muscle thickness; LF = loaded-force; FV = force-velocity; TF = temporal-force; LCMJ = loaded countermovement jump; CMJ = countermovement jump; SJ = squat jump; LSJ = loaded squat jump; RFD = rate of force development; TtPF = time to peak force; FVA = force-velocity area; EccAccT = eccentric acceleration time; CT = concentric time; TtPP = time to peak power.

VL PA was associated with eccentric acceleration time across CMJ and LCMJ, and with concentric time across squat-jump conditions (*r* = 0.52 [0.06, 0.80] to 0.61 [0.18, 0.84], *p* < 0.01 to 0.03). Associations with time to peak power were of similar moderate magnitude for SJ (VL PA: *r* = 0.49 [0.02, 0.79], *p* = 0.04) and LSJ (VL PA: *r* = 0.47 [−0.02, 0.77], *p* = 0.06), although the LSJ association did not reach the conventional significance threshold. VL MT was associated with LSJ concentric time (*r* = 0.56 [0.10, 0.82], *p* = 0.02) and LSJ time to peak power (*r* = 0.55 [0.09, 0.81], *p* = 0.02; Figure 2; Table 5).

### 3.3. Skeletal Muscle Architecture and Transfer-Efficiency Indices

Transfer-efficiency indices showed no significant associations with skeletal muscle architecture, except the LCMJ/LSJ concentric time ratio, which showed a moderate negative association with LG PA (*r* = −0.49 [−0.79, −0.01]; *p* = 0.05; Table 6).

### 3.4. Supplementary Analyses: Conventional Absolute Outcomes

Conventional absolute outcomes showed predominantly null associations with skeletal muscle architecture; of 216 correlations tested, four reached the conventional significance threshold, all involving squat-jump concentric time (Supplementary Tables S1 and S2).

## 4. Discussion

The present study aimed to investigate the associations between resting LG and VL architecture and force–time strategy under loaded and unloaded vertical jump conditions in collegiate basketball players. A secondary aim was to examine the relationships between SMA and jump-height retention and SSC transfer-efficiency indices. External loading produced very large to extremely large reductions in velocity and power outcomes and moderate to very large increases in force outcomes. LG PA was negatively associated with LCMJ RFD and positively associated with LCMJ time to peak force, in the direction predicted a priori by architectural gearing theory. VL PA was associated with the temporal organization of force expression, whereas loaded-retention and SSC transfer-efficiency indices were largely unrelated to SMA. These findings suggest that resting muscle architecture provides a structural context for the strategy through which force is expressed under load, rather than for the gross preservation of jump height under load.

### 4.1. LG Architecture and Loaded Force Development

LG PA was negatively associated with LCMJ RFD and positively associated with LCMJ time to peak force, in the direction predicted a priori by architectural gearing theory. Both associations survived FDR correction within the loaded force-development group but did not survive the pooled correction across all morphology-sensitive correlations. These associations therefore remain preliminary, and the present evidence should be regarded as hypothesis-generating rather than confirmatory. This pattern is consistent with architectural gearing theory [3,5]. A greater PA increases the physiological cross-sectional area available for maximal force production, whereas it may simultaneously constrain the effective rate of force transmission to the tendon during dynamic SSC actions. Prior in vivo evidence indicates that resting fascicle angle is associated with RFD during dynamic contractions, and that the strength of this relationship is modulated by contraction-specific gear changes [2,3]. Under the LCMJ condition, substantial external inertia may amplify this association, as the extended braking phase requires a rapid transition to concentric force expression. The positive association between LG PA and time to peak force is consistent with this interpretation, as athletes with greater LG PA tended to require longer to reach peak force under load, irrespective of absolute force capacity. Although static ultrasonography cannot directly assess dynamic gearing mechanisms, the survival of these associations through within-group correction, combined with their theoretically predicted pattern, supports their interpretation as the strongest signal in the dataset. These associations are therefore best interpreted as structurally consistent with gearing theory rather than as mechanistic confirmation. The magnitude and direction of these associations provide a structural context for interpreting individual differences in loaded explosive force expression [14,15,29].

### 4.2. VL Architecture and Temporal Force-Expression

VL PA was associated with eccentric acceleration time across CMJ and LCMJ and with concentric time across squat-jump conditions. This pattern is distinct from the LG RFD pattern and is consistent with the different mechanical roles of these muscles during vertical jumping. The LG is a biarticular muscle with relatively longer fascicles that operate closer to optimal shortening velocity during explosive tasks [3,30], which may enable a direct architectural influence on the rate of force development. In contrast, the unipennate architecture and typically greater PA of the VL may predispose it to shape the temporal distribution of force during the concentric phase [12], particularly in squat-jump conditions where pre-stretch augmentation is absent. Previous research in female athletes reported no significant associations between VL architecture and eccentric RFD during jumping [9]. In the present study, the associations were observed specifically with eccentric acceleration time, which suggests that VL architecture relates to the temporal modulation of force during the descent phase rather than its rate. A greater VL PA is also associated with reduced fascicle shortening velocity, such that athletes with this structural profile favor a more extended eccentric phase [31,32]. These associations were of large magnitude but did not survive FDR correction at either level and are interpreted as exploratory.

### 4.3. Applied Implications for Basketball Monitoring

At the practical monitoring level, two basketball players achieving identical unloaded jump heights can operate under substantially different structural conditions that become relevant under high-inertia game demands. An athlete with greater LG PA who produces adequate force during uncontested jumps may show attenuated force expression during resisted, contested actions where external inertia demands are elevated. Such differences are not apparent from conventional unloaded jump-height monitoring alone [14,29,33]. Sport-specific differences in gastrocnemius architectural profiles have been observed among elite athletes [34], and these structural characteristics may have functional relevance for loaded force-development strategies in basketball. The integration of LG architectural assessment with loaded force–time profiling warrants further investigation as a means of characterizing individual force-development strategies in this population [34]. These applied considerations derive from an exploratory sample of seventeen players. They are therefore offered as directions for practice rather than as established monitoring recommendations.

### 4.4. Loaded Retention and SSC Transfer

Transfer-efficiency indices were not significantly associated with skeletal muscle architecture, except for the LCMJ/LSJ concentric time ratio. The gross preservation of jump height across loading conditions may depend more on pre-activation timing, motor-unit recruitment thresholds, and the elimination of musculotendinous slack, processes primarily under central neural control and structurally independent of resting architectural morphology [16,17]. This interpretation is consistent with loaded ballistic research indicating that peak quadriceps activation declines with increasing external load, with increases in integrated electromyography attributable to longer concentric phase duration rather than greater activation intensity [19]. Between-participant variability in mechanical output under load has likewise been attributed to differences in fiber-type distribution, motor-unit recruitment, and training background, with limited dependence on static muscle morphology [19]. The loaded-retention construct therefore isolates a reliable performance quality (ICC = 0.87–0.88, Table 3) that conventional architecture-based or unloaded jump-height monitoring cannot capture. Its validity has not yet been established and requires confirmation in larger samples. The negative association between LG PA and the LCMJ/LSJ concentric time ratio suggests that athletes with greater LG PA may derive a smaller temporal benefit from the countermovement under load. This association emerged in the temporal dimension of SSC transfer, a distinction not captured by single jump-height ratios such as the eccentric utilization ratio [16,17,27]. These findings indicate that resting SMA is associated with the rate and temporal organization of force expression, rather than with the gross preservation of jump-height output under load.

### 4.5. Limitations and Future Research Direction

Although this is the first study to examine the associations between resting muscle architecture and force–time strategy under externally loaded conditions in collegiate basketball players, the findings should be interpreted with several limitations in mind. First, the sample size was small, which limited the statistical power to detect weaker but mechanistically relevant associations. Furthermore, the data-driven selection of variables based on their statistical reliability within this specific cohort introduces a potential selection bias, meaning these exploratory associations may exhibit sample specificity and require independent replication. Second, the exploratory nature of the analyses, combined with the pooled false discovery rate correction across 60 morphology-sensitive correlations, retained only the two LG PA associations with RFD and time to peak force. The remaining associations, including the coherent pattern of VL PA with temporal outcomes, are therefore hypothesis-generating and require confirmation in adequately powered studies. Third, a single external load of 20 kg was used; whether the observed architectural associations scale across a load spectrum remains to be examined. Fourth, static B-mode ultrasonography cannot directly assess dynamic gearing mechanisms during the stretch–shortening cycle; real-time imaging of fascicle behavior during loaded jumping would provide a more direct test of the architectural gearing predictions. Fifth, architectural assessment was restricted to the VL and LG, so the findings characterize only part of the lower-limb extensor chain. Sixth, fascicle length was estimated geometrically rather than measured directly and should be interpreted accordingly.

Future research should therefore examine these associations in larger and adequately powered samples. Furthermore, assessing a spectrum of external loads would clarify whether the observed architectural associations scale with load intensity. Given the limitations of static ultrasonography, real-time fascicle imaging during loaded jumping is recommended to directly test the gearing predictions. Finally, the inclusion of additional lower-limb extensors, alongside longitudinal designs, would extend structural coverage and determine whether loaded retention effectively tracks training adaptation.

## 5. Conclusions

This is the first study to examine the associations between resting skeletal muscle architecture of the VL and LG and force–time strategy across loaded and unloaded vertical jump conditions in collegiate basketball players. LG PA was associated with loaded RFD and time to peak force in the direction predicted a priori by architectural gearing theory. VL PA was associated with the temporal organization of force expression, whereas loaded-retention and SSC-transfer indices were largely unrelated to architecture. These findings indicate that resting muscle architecture provides a structural context for the strategy through which force is expressed under load, rather than for the gross preservation of jump height. Consequently, integrating architectural assessment with loaded force–time profiling warrants further investigation as a means of characterizing individual force-development strategies.

## Figures and Tables

**Table 1:** Definitions of force–time variable groups and transfer-efficiency indices used in the present study.

Variable Group	Variable	Test Condition	Definition
Part A. Force–time variable groups			
Loaded force development	Rate of force development (RFD)	LCMJ	Peak rate of increase in vertical ground reaction force during the concentric phase under external load (N·s^−1^)
	Time to peak force	LCMJ	Duration from concentric phase onset to the instant of peak force under external load (s)
Force-velocity expression	Force-velocity area	CMJ, LCMJ	Area under the force–velocity curve during the concentric phase; reflects the mechanical bandwidth of force production across the velocity spectrum [13]
Temporal force-expression	Eccentric acceleration time	CMJ, LCMJ	Duration of the eccentric acceleration sub-phase, from movement onset to peak negative (downward) velocity (s)
	Concentric time	SJ, LSJ	Total duration of the concentric phase in squat-jump conditions, where pre-stretch augmentation is absent (s)
	Time to peak power	SJ, LSJ	Duration from concentric phase onset to the instant of peak power output in squat-jump conditions (s)
Part B. Transfer-efficiency indices			
Loaded retention	LCMJ/CMJ height ratio	LCMJ vs. CMJ	Percentage of unloaded CMJ height preserved under a 20 kg external load; reflects the capacity to maintain jump-height output under a fixed external load (%)
	LSJ/SJ height ratio	LSJ vs. SJ	Percentage of unloaded SJ height preserved under a 20 kg external load (%)
SSC transfer (unloaded)	CMJ/SJ ratios	CMJ vs. SJ	Countermovement-to-squat-jump ratio under unloaded conditions; reflects the pre-stretch benefit conferred by the stretch–shortening cycle. Corresponds to the EUR construct [16,17,26,27] (%)
SSC transfer (loaded)	LCMJ/LSJ ratios	LCMJ vs. LSJ	Countermovement-to-squat-jump ratio under loaded conditions; reflects SSC efficiency when external inertia is present (%)

SSC = stretch–shortening cycle; LCMJ = loaded countermovement jump; CMJ = countermovement jump; SJ = squat jump; LSJ = loaded squat jump.

**Table 2:** Descriptive and reliability statistics for selected morphology-sensitive force–time outcomes.

Variable Group	Test	Variable	Mean ± SD	CV (%)	ICC [95% CI]	SEM
LF-development	LCMJ	RFD (N·s^−1^)	5538.20 ± 1478.37	9.00	0.94 [0.77, 0.98]	498.34
	LCMJ	TtPF (s)	0.22 ± 0.08	4.25	0.99 [0.79, 1.00]	0.01
FV-expression	CMJ	FVA (N·m·s^−1^)	2482.67 ± 393.41	6.87	0.90 [0.65, 0.99]	170.49
	LCMJ	FVA (N·m·s^−1^)	2458.02 ± 488.77	7.73	0.93 [0.82, 0.96]	189.93
TF-expression	CMJ	EccAccT (s)	0.31 ± 0.02	4.69	0.84 [0.51, 0.95]	0.01
	LCMJ	EccAccT (s)	0.29 ± 0.02	5.33	0.80 [0.13, 0.93]	0.02
	SJ	CT (s)	0.32 ± 0.04	4.34	0.92 [0.73, 0.98]	0.01
	LSJ	CT (s)	0.36 ± 0.04	3.75	0.93 [0.76, 0.98]	0.01
	SJ	TtPP (s)	0.26 ± 0.04	5.58	0.92 [0.74, 0.98]	0.01
	LSJ	TtPP (s)	0.30 ± 0.04	5.64	0.89 [0.62, 0.97]	0.02

Values are mean ± SD. CV = coefficient of variation; ICC = intraclass correlation coefficient; CI = confidence interval; SEM = standard error of measurement. LCMJ = loaded countermovement jump; CMJ = countermovement jump; SJ = squat jump; LSJ = loaded squat jump; LF = loaded-force; FV = force-velocity; TF = temporal-force; RFD = rate of force development; TtPF = time to peak force; FVA = force-velocity area; EccAccT = eccentric acceleration time; CT = concentric time; TtPP = time to peak power.

**Table 3:** Descriptive and reliability statistics for selected loaded-retention and SSC-transfer indices.

Domain	Test	Variable	Mean ± SD	CV (%)	ICC [95% CI]	SEM
Loaded retention	LCMJ/CMJ	JH ratio (%)	75.77 ± 4.68	2.76	0.88 [0.69, 0.95]	2.09
Loaded retention	LSJ/SJ	JH ratio (%)	79.39 ± 5.37	3.46	0.87 [0.51, 0.93]	2.75
SSC transfer (unloaded)	CMJ/SJ	JH ratio (%)	121.85 ± 7.82	3.12	0.89 [0.67, 0.95]	3.80
SSC transfer (loaded)	LCMJ/LSJ	JH ratio (%)	116.33 ± 7.19	3.63	0.83 [0.64, 0.91]	4.22
SSC transfer (unloaded)	CMJ/SJ	PF ratio (%)	111.08 ± 14.25	4.61	0.94 [0.85, 0.98]	5.12
SSC transfer (unloaded)	CMJ/SJ	CT ratio (%)	82.62 ± 10.93	7.26	0.84 [0.55, 0.96]	6.00
SSC transfer (loaded)	LCMJ/LSJ	PF ratio (%)	108.14 ± 14.41	3.56	0.96 [0.91, 0.99]	3.85
SSC transfer (loaded)	LCMJ/LSJ	CT ratio (%)	83.65 ± 9.75	5.24	0.90 [0.71, 0.97]	4.38

Values are mean ± SD. Ratios were calculated at the paired-trial level as numerator/denominator × 100. Loaded retention includes LCMJ/CMJ and LSJ/SJ. SSC transfer includes CMJ/SJ and LCMJ/LSJ. For SSC indices, values above 100% indicate that the countermovement condition exceeded the squat-jump condition; the corresponding gain can be obtained by subtracting 100%. CV = coefficient of variation; ICC = intraclass correlation coefficient; CI = confidence interval; SEM = standard error of measurement; SSC = stretch–shortening cycle; LCMJ = loaded countermovement jump; CMJ = countermovement jump; LSJ = loaded squat jump; SJ = squat jump; JH = jump height; PF = peak force; CT = concentric time.

**Table 4:** Paired comparisons between loaded and unloaded jump conditions.

Comparison	Variable	Loaded M ± SD	Unloaded M ± SD	Mean Difference	Difference (%)	t	*p*	Hedges’ *g*
LCMJ-CMJ	JH (m)	0.33 ± 0.04	0.43 ± 0.06	−0.11	−24.24	−14.84	<0.01	−3.43
LCMJ-CMJ	FT (s)	0.51 ± 0.03	0.59 ± 0.04	−0.08	−13.00	−16.87	<0.01	−3.90
LCMJ-CMJ	PF (N·kg^−1^)	25.53 ± 1.64	24.90 ± 2.14	0.63	2.79	2.73	0.02	0.63
LCMJ-CMJ	F@PP (N·kg^−1^)	23.36 ± 1.97	21.58 ± 1.62	1.79	8.23	11.62	<0.01	2.68
LCMJ-CMJ	PV (m·s^−1^)	2.56 ± 0.16	2.90 ± 0.20	−0.34	−11.66	−17.42	<0.01	−4.02
LCMJ-CMJ	TOV (m·s^−1^)	2.42 ± 0.17	2.78 ± 0.21	−0.36	−12.89	−17.94	<0.01	−4.14
LCMJ-CMJ	MCF (N·kg^−1^)	22.24 ± 1.67	21.12 ± 1.64	1.11	5.32	9.81	<0.01	2.27
LCMJ-CMJ	MCP (W·kg^−1^)	13.45 ± 2.20	16.63 ± 2.91	−3.17	−18.75	−10.75	<0.01	−2.48
LSJ-SJ	JH (m)	0.28 ± 0.03	0.35 ± 0.04	−0.07	−20.62	−13.70	<0.01	−3.16
LSJ-SJ	FT (s)	0.48 ± 0.03	0.54 ± 0.03	−0.06	−10.95	−14.53	<0.01	−3.36
LSJ-SJ	PF (N·kg^−1^)	24.01 ± 3.53	22.69 ± 2.87	1.33	5.77	3.48	<0.01	0.80
LSJ-SJ	F@PP (N·kg^−1^)	22.92 ± 3.58	21.48 ± 2.93	1.43	6.74	3.17	0.01	0.73
LSJ-SJ	PV (m·s^−1^)	2.14 ± 0.39	2.43 ± 0.41	−0.29	−11.40	−4.42	<0.01	−1.02
LSJ-SJ	TOV (m·s^−1^)	1.85 ± 0.38	2.17 ± 0.41	−0.32	−14.16	−5.35	<0.01	−1.24
LSJ-SJ	MCF (N·kg^−1^)	19.34 ± 2.05	17.77 ± 1.73	1.57	8.83	7.05	<0.01	1.63
LSJ-SJ	MCP (W·kg^−1^)	8.15 ± 3.12	9.81 ± 3.26	−1.66	−15.80	−3.59	<0.01	−0.83

Values are M ± SD. Paired-samples *t* tests compared the loaded against the unloaded conditions. Difference = loaded − unloaded. Difference (%) was calculated for each athlete as (loaded − unloaded)/unloaded × 100 and then averaged. LCMJ = loaded countermovement jump; CMJ = countermovement jump; LSJ = loaded squat jump; SJ = squat jump; JH = jump height; FT = flight time; PF = peak force; F@PP = force at peak power; PV = peak velocity; TOV = take-off velocity; MCF = mean concentric force; MCP = mean concentric power.

**Table 5:** Correlations between selected morphology-sensitive force–time outcomes and ultrasound-derived muscle architecture.

Variable Group	Test	Variable	LG PA	LG MT	LG FL	VL PA	VL MT	VL FL
LF-development	LCMJ	RFD (N·s^−1^)	−0.68 [−0.87, −0.30] *^†^	−0.58 [−0.83, −0.13] *	−0.14 [−0.58, 0.37]	0.07 [−0.43, 0.53]	0.05 [−0.44, 0.52]	−0.06 [−0.53, 0.43]
	LCMJ	TtPF (s)	0.68 [.29, 0.87] *^†^	0.53 [.07, 0.81] *	0.05 [−0.44, 0.52]	−0.08 [−0.54, 0.42]	0.05 [−0.44, 0.52]	0.21 [−0.30, 0.63]
FV-expression	CMJ	FVA (N·m·s^−1^)	−0.58 [−0.83, −0.14] *	−0.38 [−0.73, 0.12]	0.00 [−0.48, 0.48]	−0.22 [−0.64, 0.29]	0.05 [−0.44, 0.52]	0.32 [−0.19, 0.69]
	LCMJ	FVA (N·m·s^−1^)	−0.50 [−0.79, −0.03] *	−0.44 [−0.76, 0.06]	−0.15 [−0.59, 0.36]	−0.15 [−0.59, 0.35]	0.05 [−0.44, 0.52]	0.23 [−0.28, 0.64]
TF-expression	CMJ	EccAccT (s)	−0.12 [−0.57, 0.39]	0.06 [−0.43, 0.53]	0.18 [−0.33, 0.61]	0.54 [0.08, 0.81] *	0.20 [−0.31, 0.62]	−0.55 [−0.81, −0.09] *
	LCMJ	EccAccT (s)	−0.17 [−0.60, 0.34]	−0.17 [−0.60, 0.34]	−0.08 [−0.54, 0.42]	0.61 [0.18, 0.84] *	0.31 [−0.20, 0.69]	−0.48 [−0.78, 0.01]
	SJ	CT (s)	0.42 [−0.08, 0.75]	0.15 [−0.35, 0.59]	−0.19 [−0.61, 0.32]	0.58 [0.14, 0.83] *	0.44 [−0.05, 0.76]	−0.22 [−0.63, 0.29]
	LSJ	CT (s)	0.50 [0.02, 0.79] *	0.30 [−0.21, 0.68]	−0.10 [−0.55, 0.40]	0.52 [0.06, 0.80] *	0.56 [0.10, 0.82] *	0.02 [−0.47, 0.49]
	SJ	TtPP (s)	0.43 [−0.06, 0.75]	0.15 [−0.36, 0.59]	−0.21 [−0.63, 0.30]	0.49 [.02, 0.79] *	0.31 [−0.20, 0.69]	0.26 [−0.66, 0.25]
	LSJ	TtPP (s)	0.50 [0.03, 0.79] *	0.34 [−0.17, 0.71]	−0.05 [−0.52, 0.44]	0.47 [−0.02, 0.77]	0.55 [.09, 0.81] *	0.08 [−0.42, 0.54]

Values are Pearson *r* [95% CI]. LG = lateral gastrocnemius; VL = vastus lateralis; PA = pennation angle; MT = muscle thickness; FL = fascicle length; LCMJ = loaded countermovement jump; CMJ = countermovement jump; SJ = squat jump; LSJ = loaded squat jump; LF = loaded-force; FV = force-velocity; TF = temporal-force; RFD = rate of force development; TtPF = time to peak force; FVA = force-velocity area; EccAccT = eccentric acceleration time; CT = concentric time; TtPP = time to peak power. * *p* < 0.05. ^†^ Remained significant after within-group Benjamini–Hochberg FDR correction.

**Table 6:** Correlations between transfer-efficiency indices and ultrasound-derived muscle architecture.

Domain	Pair	Variable	LG PA	LG MT	LG FL	VL PA	VL MT	VL FL
Loaded retention	LCMJ/CMJ	JH ratio (%)	0.02 [−0.46, 0.50]	−0.13 [−0.58, 0.37]	−0.23 [−0.64, 0.28]	0.27 [−0.24, 0.66]	0.16 [−0.35, 0.59]	−0.22 [−0.63, 0.29]
Loaded retention	LSJ/SJ	JH ratio (%)	−0.10 [−0.56, 0.40]	−0.43 [−0.75, 0.07]	−0.47 [−0.78, 0.01]	−0.04 [−0.51, 0.45]	−0.07 [−0.53, 0.42]	−0.06 [−0.52, 0.44]
SSC transfer (unloaded)	CMJ/SJ	JH ratio (%)	−0.20 [−0.62, 0.31]	−0.07 [−0.53, 0.43]	0.12 [−0.38, 0.57]	−0.10 [−0.56, 0.40]	−0.24 [−0.65, 0.27]	−0.19 [−0.61, 0.32]
SSC transfer (loaded)	LCMJ/LSJ	JH ratio (%)	−0.07 [−0.53, 0.43]	0.25 [−0.27, 0.65]	0.39 [−0.11, 0.73]	0.19 [−0.32, 0.61]	−0.03 [−0.51, 0.45]	−0.35 [−0.71, 0.15]
SSC transfer (unloaded)	CMJ/SJ	PF ratio (%)	−0.11 [−0.56, 0.39]	0.06 [−0.44, 0.52]	0.19 [−0.32, 0.62]	0.26 [−0.25, 0.66]	0.24 [−0.27, 0.65]	−0.07 [−0.53, 0.43]
SSC transfer (unloaded)	CMJ/SJ	CT ratio (%)	−0.47 [−0.78, 0.01]	−0.03 [−0.50, 0.46]	0.40 [−0.10, 0.74]	−0.29 [−0.68, 0.22]	−0.22 [−0.64, 0.29]	0.05 [−0.44, 0.52]
SSC transfer (loaded)	LCMJ/LSJ	PF ratio (%)	0.08 [−0.42, 0.54]	−0.02 [−0.49, 0.47]	−0.09 [−0.54, 0.41]	0.26 [−0.26, 0.66]	0.17 [−0.33, 0.60]	−0.12 [−0.57, 0.38]
SSC transfer (loaded)	LCMJ/LSJ	CT ratio (%)	−0.49 [−0.79, −0.01] *	−0.16 [−0.60, 0.35]	0.27 [−0.24, 0.66]	−0.27 [−0.66, 0.25]	−0.38 [−0.73, 0.13]	−0.19 [−0.61, 0.32]

Values are Pearson *r* [95% CI]. LG = lateral gastrocnemius; VL = vastus lateralis; PA = pennation angle; MT = muscle thickness; FL = fascicle length. LCMJ = loaded countermovement jump; CMJ = countermovement jump; SJ = squat jump; LSJ = loaded squat jump; SSC = stretch–shortening cycle; JH = jump height; PF = peak force; CT = concentric time. * *p* < 0.05; no association remained significant after FDR correction.

## Data Availability

The original contributions presented in the study are included in the article; further inquiries can be directed to the corresponding author.

## Supplementary Materials

The following supporting information can be downloaded at: https://www.mdpi.com/article/10.3390/jfmk11030246/s1.
