# Supplementary material for: Exploratory Analysis of Skeletal Muscle Architecture and Force–Time Strategy Under External Load in Collegiate Basketball Players
Source: J Funct Morphol Kinesiol. 2026 Jun 24;11(3):246. doi: 10.3390/jfmk11030246 (PMC13397912; doi:10.3390/jfmk11030246)
Supplement: Supplementary file 1 [file jfmk-11-00246-s001.zip › jfmk-4352632-supplementary.pdf]

Supplementary Table S1. Descriptive and reliability statistics for core absolute jump outcomes

Test	Variable	Mean ± SD	CV (%)	ICC [95% CI]	SEM
CMJ	JH (m)	0.43 ± 0.06	1.82	0.99 [0.98, 1.00]	0.01
CMJ	FT (s)	0.59 ± 0.04	0.92	0.99 [0.98, 1.00]	0.01
CMJ	PF (N·kg⁻¹)	24.90 ± 2.14	1.90	0.98 [0.95, 0.99]	0.47
CMJ	F@PP (N·kg⁻¹)	21.58 ± 1.62	1.66	0.98 [0.94, 0.99]	0.36
CMJ	PV (m·s⁻¹)	2.90 ± 0.20	1.08	0.99 [0.96, 1.00]	0.03
CMJ	TOV (m·s⁻¹)	2.78 ± 0.21	1.29	0.99 [0.96, 1.00]	0.04
CMJ	MCF (N·kg⁻¹)	21.12 ± 1.64	1.88	0.97 [0.89, 0.98]	0.40
CMJ	MCP (W·kg⁻¹)	16.63 ± 2.91	3.72	0.98 [0.94, 0.99]	0.62
CMJ	CT (s)	0.26 ± 0.03	4.47	0.90 [0.67, 0.93]	0.01
LCMJ	JH (m)	0.33 ± 0.04	1.75	0.99 [0.97, 0.99]	0.01
LCMJ	FT (s)	0.51 ± 0.03	0.88	0.99 [0.97, 0.99]	0.00
LCMJ	PF (N·kg⁻¹)	25.53 ± 1.64	2.43	0.93 [0.76, 0.97]	0.62
LCMJ	F@PP (N·kg⁻¹)	23.36 ± 1.97	1.57	0.98 [0.95, 0.99]	0.37
LCMJ	PV (m·s⁻¹)	2.56 ± 0.16	1.03	0.98 [0.96, 0.99]	0.03
LCMJ	TOV (m·s⁻¹)	2.42 ± 0.17	1.10	0.99 [0.96, 0.99]	0.03
LCMJ	MCF (N·kg⁻¹)	22.24 ± 1.67	2.32	0.96 [0.78, 0.99]	0.52
LCMJ	MCP (W·kg⁻¹)	13.45 ± 2.20	5.73	0.94 [0.78, 0.99]	0.77
LCMJ	CT (s)	0.30 ± 0.02	4.28	0.84 [0.36, 0.96]	0.01
SJ	JH (m)	0.35 ± 0.04	1.66	0.99 [0.98, 1.00]	0.01
SJ	FT (s)	0.54 ± 0.03	0.86	0.99 [0.97, 0.99]	0.00
SJ	PF (N·kg⁻¹)	22.69 ± 2.87	3.80	0.96 [0.89, 0.98]	0.86
SJ	F@PP (N·kg⁻¹)	21.48 ± 2.93	3.48	0.97 [0.92, 0.99]	0.75
SJ	PV (m·s⁻¹)	2.43 ± 0.41	4.40	0.97 [0.91, 0.99]	0.11
SJ	TOV (m·s⁻¹)	2.17 ± 0.41	5.44	0.95 [0.89, 0.98]	0.12
SJ	MCF (N·kg⁻¹)	17.77 ± 1.73	2.82	0.96 [0.89, 0.98]	0.50
SJ	MCP (W·kg⁻¹)	9.81 ± 3.26	9.21	0.96 [0.90, 0.99]	0.90
SJ	CT (s)	0.32 ± 0.04	4.34	0.92 [0.73, 0.98]	0.01
LSJ	JH (m)	0.28 ± 0.03	3.13	0.97 [0.92, 0.98]	0.01
LSJ	FT (s)	0.48 ± 0.03	1.57	0.97 [0.92, 0.98]	0.01
LSJ	PF (N·kg⁻¹)	24.01 ± 3.53	3.32	0.98 [0.93, 0.99]	0.80
LSJ	F@PP (N·kg⁻¹)	22.92 ± 3.58	2.94	0.98 [0.95, 0.99]	0.67
LSJ	PV (m·s⁻¹)	2.14 ± 0.39	5.59	0.95 [0.90, 0.98]	0.12
LSJ	TOV (m·s⁻¹)	1.85 ± 0.38	8.43	0.92 [0.81, 0.97]	0.16
LSJ	MCF (N·kg⁻¹)	19.34 ± 2.05	2.21	0.98 [0.95, 0.99]	0.43
LSJ	MCP (W·kg⁻¹)	8.15 ± 3.12	9.00	0.97 [0.94, 0.99]	0.73
LSJ	CT (s)	0.36 ± 0.04	3.75	0.93 [0.76, 0.98]	0.01

Note. Values are mean ± SD. Force and power variables were standardized to body mass across the 17 athletes and are reported as N·kg⁻¹ or W·kg⁻¹ as appropriate. Force at peak power represents the force value at the instant of peak power and is therefore reported in N·kg⁻¹. CV = coefficient of variation; ICC = intraclass correlation coefficient; CI = confidence interval; SEM = standard error of measurement; CMJ = countermovement jump; LCMJ = loaded countermovement jump; SJ = squat jump; LSJ = loaded squat jump; JH = jump height; FT = flight time; PF = peak force; F@PP = force at peak power; PV = peak velocity; TOV = take-off velocity; MCF = mean concentric force; MCP = mean concentric power; CT = concentric time.

Supplementary Table S2. Correlations between reliable absolute jump outcomes and ultrasound-derived muscle architecture

Panel A. Lateral gastrocnemius architecture

Test	Variable	LG PA	LG MT	LG FL
CMJ	JH (m)	-.19 [-.61, .32]	-.14 [-.58, .37]	-.04 [-.51, .45]
CMJ	FT (s)	-.18 [-.61, .32]	-.14 [-.58, .36]	-.04 [-.51, .45]
CMJ	PF (N·kg⁻¹)	-.22 [-.63, .29]	-.05 [-.52, .44]	.14 [-.36, .58]
CMJ	F@PP (N·kg⁻¹)	-.04 [-.51, .45]	-.11 [-.56, .39]	-.15 [-.59, .36]
CMJ	PV (m·s⁻¹)	-.08 [-.54, .41]	.02 [-.46, .50]	.08 [-.42, .54]
CMJ	TOV (m·s⁻¹)	-.07 [-.53, .42]	-.01 [-.49, .47]	.02 [-.46, .50]
CMJ	MCF (N·kg⁻¹)	.02 [-.47, .50]	-.09 [-.54, .41]	-.15 [-.59, .36]
CMJ	MCP (W·kg⁻¹)	.00 [-.48, .48]	-.05 [-.52, .44]	-.08 [-.54, .41]
CMJ	CT (s)	-.22 [-.64, .29]	.09 [-.40, .55]	.32 [-.19, .69]
LCMJ	JH (m)	-.21 [-.63, .30]	-.23 [-.64, .28]	-.16 [-.59, .35]
LCMJ	FT (s)	-.20 [-.62, .31]	-.23 [-.64, .28]	-.16 [-.60, .35]
LCMJ	PF (N·kg⁻¹)	-.14 [-.58, .36]	-.13 [-.58, .37]	-.05 [-.52, .44]
LCMJ	F@PP (N·kg⁻¹)	.04 [-.45, .51]	.06 [-.44, .52]	.01 [-.48, .49]
LCMJ	PV (m·s⁻¹)	.01 [-.48, .48]	.05 [-.44, .52]	.02 [-.46, .50]
LCMJ	TOV (m·s⁻¹)	.00 [-.48, .48]	.02 [-.46, .50]	-.01 [-.49, .47]
LCMJ	MCF (N·kg⁻¹)	.03 [-.45, .51]	-.05 [-.52, .44]	-.12 [-.56, .39]
LCMJ	MCP (W·kg⁻¹)	.04 [-.45, .51]	-.02 [-.50, .46]	-.09 [-.55, .40]
LCMJ	CT (s)	-.16 [-.60, .35]	.09 [-.41, .55]	.27 [-.24, .67]
SJ	JH (m)	-.11 [-.56, .39]	-.13 [-.57, .38]	-.11 [-.56, .39]
SJ	FT (s)	-.11 [-.56, .39]	-.13 [-.57, .38]	-.11 [-.56, .39]
SJ	PF (N·kg⁻¹)	-.06 [-.53, .43]	-.10 [-.55, .40]	-.09 [-.55, .41]
SJ	F@PP (N·kg⁻¹)	-.03 [-.50, .46]	-.11 [-.56, .40]	-.13 [-.58, .37]
SJ	PV (m·s⁻¹)	.23 [-.29, .64]	.02 [-.46, .50]	-.20 [-.62, .31]
SJ	TOV (m·s⁻¹)	.20 [-.31, .62]	.00 [-.48, .48]	-.21 [-.63, .30]
SJ	MCF (N·kg⁻¹)	-.07 [-.53, .43]	-.06 [-.53, .43]	-.04 [-.51, .45]
SJ	MCP (W·kg⁻¹)	.06 [-.43, .52]	-.04 [-.51, .45]	-.13 [-.58, .37]
SJ	CT (s)	.42 [-.08, .75]	.15 [-.35, .59]	-.19 [-.61, .32]
LSJ	JH (m)	-.19 [-.61, .32]	-.36 [-.72, .15]	-.35 [-.71, .16]
LSJ	FT (s)	-.17 [-.60, .33]	-.36 [-.71, .15]	-.36 [-.71, .15]
LSJ	PF (N·kg⁻¹)	-.14 [-.58, .37]	-.06 [-.52, .43]	.04 [-.45, .51]
LSJ	F@PP (N·kg⁻¹)	-.15 [-.59, .36]	-.05 [-.52, .44]	.05 [-.44, .52]
LSJ	PV (m·s⁻¹)	-.04 [-.51, .45]	-.03 [-.50, .46]	-.03 [-.50, .46]
LSJ	TOV (m·s⁻¹)	.03 [-.46, .50]	.02 [-.47, .49]	-.03 [-.51, .45]
LSJ	MCF (N·kg⁻¹)	-.22 [-.64, .29]	-.14 [-.58, .36]	.01 [-.47, .49]
LSJ	MCP (W·kg⁻¹)	-.19 [-.62, .32]	-.16 [-.60, .35]	-.06 [-.52, .43]
LSJ	CT (s)	.50 [.02, .79]*	.30 [-.21, .68]	-.10 [-.55, .40]

Panel B. Vastus lateralis architecture

Test	Variable	VL PA	VL MT	VL FL
CMJ	JH (m)	-.09 [-.55, .41]	.08 [-.42, .54]	.20 [-.31, .62]
CMJ	FT (s)	-.10 [-.55, .40]	.07 [-.42, .54]	.21 [-.30, .63]
CMJ	PF (N·kg⁻¹)	-.13 [-.57, .38]	-.15 [-.59, .36]	-.05 [-.52, .44]
CMJ	F@PP (N·kg⁻¹)	-.22 [-.63, .29]	-.10 [-.55, .40]	.17 [-.34, .60]
CMJ	PV (m·s⁻¹)	-.10 [-.55, .40]	-.03 [-.50, .46]	.08 [-.42, .54]
CMJ	TOV (m·s⁻¹)	-.06 [-.53, .43]	.02 [-.47, .49]	.08 [-.41, .54]
CMJ	MCF (N·kg⁻¹)	-.22 [-.63, .29]	-.15 [-.59, .35]	.11 [-.39, .56]
CMJ	MCP (W·kg⁻¹)	-.17 [-.60, .34]	-.10 [-.56, .40]	.11 [-.39, .56]
CMJ	CT (s)	.16 [-.35, .59]	.15 [-.36, .59]	-.09 [-.54, .41]
LCMJ	JH (m)	.02 [-.46, .50]	.16 [-.34, .60]	.13 [-.37, .57]
LCMJ	FT (s)	.02 [-.46, .50]	.16 [-.35, .60]	.13 [-.38, .57]
LCMJ	PF (N·kg⁻¹)	-.23 [-.64, .28]	-.19 [-.61, .32]	.06 [-.43, .53]
LCMJ	F@PP (N·kg⁻¹)	-.27 [-.67, .24]	-.21 [-.63, .30]	.11 [-.39, .56]
LCMJ	PV (m·s⁻¹)	-.02 [-.50, .47]	.04 [-.45, .51]	.03 [-.46, .50]
LCMJ	TOV (m·s⁻¹)	.00 [-.48, .48]	.07 [-.43, .53]	.05 [-.44, .52]
LCMJ	MCF (N·kg⁻¹)	-.26 [-.66, .25]	-.19 [-.61, .32]	.12 [-.38, .57]
LCMJ	MCP (W·kg⁻¹)	-.17 [-.60, .34]	-.09 [-.55, .41]	.11 [-.39, .56]
LCMJ	CT (s)	.21 [-.30, .63]	.13 [-.37, .58]	-.19 [-.61, .32]
SJ	JH (m)	-.06 [-.52, .44]	.20 [-.31, .62]	.32 [-.19, .70]
SJ	FT (s)	-.06 [-.52, .44]	.21 [-.30, .63]	.33 [-.18, .70]
SJ	PF (N·kg⁻¹)	-.34 [-.70, .17]	-.34 [-.71, .17]	.02 [-.47, .50]
SJ	F@PP (N·kg⁻¹)	-.28 [-.67, .23]	-.31 [-.69, .20]	-.01 [-.49, .47]
SJ	PV (m·s⁻¹)	-.04 [-.51, .45]	-.11 [-.56, .39]	-.06 [-.53, .43]
SJ	TOV (m·s⁻¹)	-.07 [-.53, .43]	-.12 [-.57, .38]	-.04 [-.51, .45]
SJ	MCF (N·kg⁻¹)	-.34 [-.71, .17]	-.33 [-.70, .18]	.03 [-.45, .51]
SJ	MCP (W·kg⁻¹)	-.23 [-.64, .28]	-.25 [-.65, .26]	.00 [-.48, .48]
SJ	CT (s)	.58 [.14, .83]*	.44 [-.05, .76]	-.22 [-.63, .29]
LSJ	JH (m)	-.08 [-.54, .42]	.18 [-.32, .61]	.32 [-.19, .69]
LSJ	FT (s)	-.08 [-.54, .42]	.18 [-.33, .61]	.31 [-.20, .69]
LSJ	PF (N·kg⁻¹)	-.30 [-.68, .21]	-.26 [-.66, .26]	.08 [-.42, .54]
LSJ	F@PP (N·kg⁻¹)	-.31 [-.69, .21]	-.24 [-.65, .27]	.11 [-.39, .56]
LSJ	PV (m·s⁻¹)	-.15 [-.59, .36]	.02 [-.46, .50]	.24 [-.27, .65]
LSJ	TOV (m·s⁻¹)	-.16 [-.60, .35]	.02 [-.47, .50]	.26 [-.25, .66]
LSJ	MCF (N·kg⁻¹)	-.36 [-.72, .14]	-.27 [-.66, .25]	.15 [-.35, .59]
LSJ	MCP (W·kg⁻¹)	-.27 [-.66, .25]	-.15 [-.59, .36]	.18 [-.33, .61]
LSJ	CT (s)	.52 [.06, .80]*	.56 [.10, .82]*	.02 [-.47, .49]

Note. Values are Pearson r [95% CI]. Force and power outcomes were standardized to body mass before correlation analysis. LG = lateral gastrocnemius; VL = vastus lateralis; PA = pennation angle; MT = muscle thickness; FL = fascicle length; CMJ = countermovement jump; LCMJ = loaded countermovement jump; SJ = squat jump; LSJ = loaded squat jump; JH = jump height; FT = flight time; PF = peak force; F@PP = force at peak power; PV = peak velocity; TOV = take-off velocity; MCF = mean concentric force; MCP = mean concentric power; CT = concentric time. $^*p < .05$.
